# NMDA Receptors Mediate Synaptic Competition in Culture

**DOI:** 10.1371/journal.pone.0024423

**Published:** 2011-09-15

**Authors:** Kevin She, Ann Marie Craig

**Affiliations:** Brain Research Centre and Department of Psychiatry, University of British Columbia, Vancouver, British Columbia, Canada; University of Washington, United States of America

## Abstract

**Background:**

Activity through NMDA type glutamate receptors sculpts connectivity in the developing nervous system. This topic is typically studied in the visual system *in vivo*, where activity of inputs can be differentially regulated, but in which individual synapses are difficult to visualize and mechanisms governing synaptic competition can be difficult to ascertain. Here, we develop a model of NMDA-receptor dependent synaptic competition in dissociated cultured hippocampal neurons.

**Methodology/Principal Findings:**

GluN1 -/- (KO) mouse hippocampal neurons lacking the essential NMDA receptor subunit were cultured alone or cultured in defined ratios with wild type (WT) neurons. The absence of functional NMDA receptors did not alter neuron survival. Synapse development was assessed by immunofluorescence for postsynaptic PSD-95 family scaffold and apposed presynaptic vesicular glutamate transporter VGlut1. Synapse density was specifically enhanced onto minority wild type neurons co-cultured with a majority of GluN1 -/- neighbour neurons, both relative to the GluN1 -/- neighbours and relative to sister pure wild type cultures. This form of synaptic competition was dependent on NMDA receptor activity and not conferred by the mere physical presence of GluN1. In contrast to these results in 10% WT and 90% KO co-cultures, synapse density did not differ by genotype in 50% WT and 50% KO co-cultures or in 90% WT and 10% KO co-cultures.

**Conclusions/Significance:**

The enhanced synaptic density onto NMDA receptor-competent neurons in minority coculture with GluN1 -/- neurons represents a cell culture paradigm for studying synaptic competition. Mechanisms involved may include a retrograde ‘reward’ signal generated by WT neurons, although in this paradigm there was no ‘punishment’ signal against GluN1 -/- neurons. Cell culture assays involving such defined circuits may help uncover the rules and mechanisms of activity-dependent synaptic competition in the developing nervous system.

## Introduction

Synaptic activity mediated by NMDA type glutamate receptors sculpts the wiring of the nervous system, regulating functional and structural connectivity [1], [2]. As studied most intensely in the developing visual system [3], activity in part via NMDA receptors controls axon and dendrite arbor development and refines connectivity. For example, in the developing tadpole retinotectal system, NMDA receptor antagonists enhance rates of axon branch addition and retraction, reduce rates of dendrite branch addition and retraction, and reduce retinotopic precision [4], [5]. NMDA receptors mediate selective connectivity by acting as calcium permeable molecular coincidence detectors gated by both ligand and voltage [6], [7]. NMDA receptors open to allow calcium entry only when glutamate release from presynaptic inputs coincides with sufficient postsynaptic depolarization to relieve magnesium block. NMDA receptor-dependent signalling cascades [8] and gene transcription [9] regulate synapse assembly, maturation, and refinement. The contribution of NMDA receptors to detect differences in patterns of activity among inputs is thought to mediate selective elimination of some inputs and retention and strengthening of other inputs. Although NMDA receptors sculpt much of the developing circuitry in the nervous system [1], [2], [4], the precise rules of competition and underlying molecular mechanisms are not well understood.

Targeted deletion of the essential GluN1 subunit results in ablation of all NMDA receptor function in mice [10] and can be used to study NMDA receptor mediated aspects of development. Whisker-related somatosensory patterning fails to develop in mice lacking GluN1 [11], or with insufficient levels of GluN1 [12] or bearing GluN1 N598R deficient in calcium permeability and in magnesium block and thus coincidence detection [13]. The altered synaptic connectivity in GluN1 deficient mice is associated with unsegregated exuberant terminal axon arbors and unoriented longer dendrite arbors in the trigeminal principal nucleus [14]. Cortex-restricted knockout of GluN1 results in a similar lack of refinement of primary somatosensory cortical dendrite arbors and thalamocortical axon arbors and loss of patterning [15], [16]. Single cell deletion of GluN2B in barrel cortex revealed cell autonomous roles of NMDA receptors in dendrite patterning [17]. There may be altered synapse density on cortical GluN1 -/- neurons, with increased spine density reported on layer IV spiny stellate cells [18] and reduced spine density reported on layer II/III pyramidal neuron basal dendrites [19].

Cell culture confers advantages of clarity in visualizing all synapses onto a given neuron and ease of manipulating neurons for assessing mechanisms. GluN1 -/- cortical or hippocampal neurons differentiate in dissociated culture, forming structural and functional synapses with apparently normal AMPA receptor mediated miniature excitatory postsynaptic currents but with reduced dendritic spine density [19], [20], [21]. Here, we test whether NMDA receptor mediated synaptic competition can be modeled in primary neuron culture. We find that GluN1 -/- and wild type hippocampal neurons develop activity-dependent differences in synapse density only when forced to compete in co-cultures of defined ratio. Based on these results, we discuss mechansims underlying NMDA receptor mediated synaptic competition involving a retrograde ‘reward’ signal.

## Results

### Hippocampal GluN1 -/- neurons exhibit normal survival and synapse density in culture

**Figure 1:**
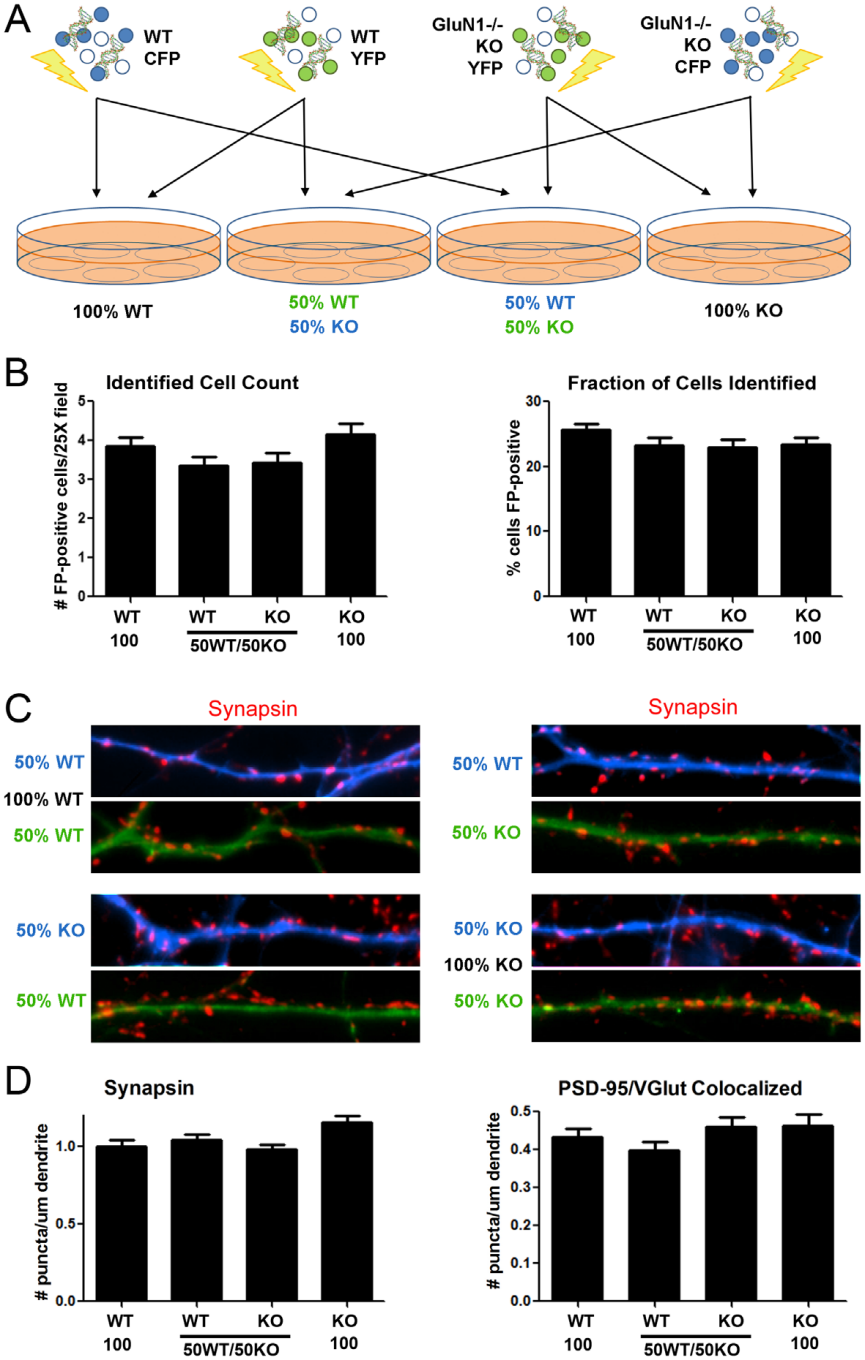
Hippocampal GluN1 -/- neurons develop normal synapse density in an equal co-culture with wild type neurons. (**A**) Hippocampal neurons from littermate wild type or GluN1 -/- mice were labeled by nucleofection and grown in co-cultures as indicated. (**B**) There was no significant difference in cell survival at 14 DIV according to genotype or culture composition. Values indicate number and percent of cells per microscope field labeled with YFP or CFP representing the indicated genotype; the 50% WT and 50% KO data come from the same coverslips of YFP WT mixed with CFP KO and of CFP WT mixed with YFP KO; ANOVA p>0.05; n = 60 from two independent experiments. (**C**) At 14 DIV, neurons were fixed and immunolabeled for synaptic markers. Neurons of defined genotype were identified by the YFP or CFP dendrite fill. Synapse density appeared similar regardless of genotype and culture composition. (**D**) There was no significant difference according to genotype or culture composition in synapsin puncta marking total synapse density or in colocalized PSD-95 and VGlut1 puncta marking excitatory synapse density; the 50% WT and 50% KO data come from the same coverslips of YFP WT mixed with CFP KO and of CFP WT mixed with YFP KO; ANOVA p>0.1; n≥50 from 3 independent experiments. All data are presented as mean ± SEM.

To test whether neurons lacking functional NMDA receptors compete less effectively for synapses in a cell culture model system, we cultured hippocampal neurons from GluN1 -/- mice (KO neurons) together with hippocampal neurons from littermate wild type mice (WT neurons). To identify neurons in mixed co-culture, WT neurons were nucleofected before plating with an expression vector for YFP and KO neurons with an expression vector for CFP, or vice versa (Fig. 1A). Transfection efficiency was on average 23.9%. Only neurons expressing YFP or CFP and thus of identified genotype were studied further in the resultant cultures. Cultures of pure WT, pure KO, and co-cultures of 50% WT 50% KO were generated such that potential effects of YFP or CFP expression or differential nucleofection would not bias results (Fig. 1A). Cultures were analyzed at 14 days in vitro (DIV).

Cell survival was assessed by counting YFP-positive and CFP-positive neurons of identified genotype and total number of neurons per field. There was no significant difference according to genotype or culture composition in numbers of surviving YFP and CFP expressing neurons, nor in the fraction of surviving neurons expressing YFP or CFP (Fig. 1B; ANOVA p>0.05). There was also no significant difference in total neuron survival among cultures of different genotype composition (ANOVA p>0.05). Thus, in these optimized low density serum-free cultures with a glial feeder layer, the absence of GluN1 does not affect hippocampal neuron survival, unlike high density cultures grown with serum [20], [22] but like *in vivo* conditions [15], [23].

Immunofluorescence for the presynaptic marker synapsin showed a similar density of input synapses onto KO neurons and WT neurons both in pure cultures and in the 50∶50 co-culture (Fig. 1C). Indeed, quantitation performed blind to genotype and culture composition revealed no significant difference in density of synapsin puncta indicating presynaptic terminals onto KO neurons or WT neurons in pure cultures or in the 50∶50 co-culture (Fig. 1D; ANOVA p>0.1). We next assessed excitatory synapses by immunolabeling for the glutamatergic postsynaptic scaffold PSD-95 family and the excitatory presynaptic vesicular glutamate transporter VGlut1. There was no significant difference according to genotype or culture composition in density of PSD-95 puncta (ANOVA p>0.1) or of PSD-95 puncta colocalized with VGlut1 marking excitatory synapses (Fig. 1D; ANOVA p>0.1). Thus, hippocampal GluN1 -/- neurons develop a steady-state synapse density equivalent to wild type neurons when cultured alone or when forced to compete in a 50∶50 mixed co-culture with wild type neurons.

### Synaptic competition occurs in mixed wild type and GluN1 -/- co-culture of defined ratio

**Figure 2:**
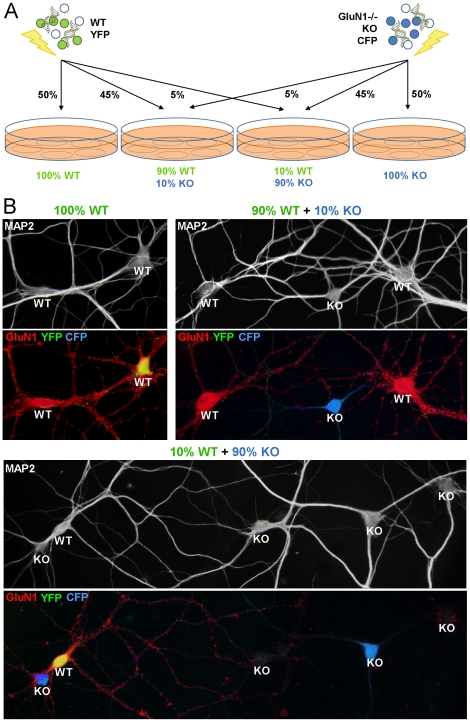
Paradigm for generating synaptic competition with unequal co-culture of GluN1 -/- and wild type neurons. (A) Hippocampal neurons from littermate wild type or GluN1 -/- mice were labeled by nucleofection and grown in co-culture. This diagram represents one form of the experiment; in the other form, the wild type neurons were labeled with CFP and GluN1 -/- neurons labeled with YFP and co-cultured in similar proportions to ensure no bias due to the nucleofected label. (B) Sample co-culture fields were immunolabeled for GluN1 and the dendrite marker MAP2. In co-cultures generated as in panel (A), all YFP neurons were confirmed immunopositive for GluN1, and CFP neurons confirmed immunonegative for GluN1, with the genotype of untransfected neurons overall as expected for the culture composition.

We next increased potential competitive pressure for synaptogenesis, reasoning that KO neurons grown in a minority with WT, or WT neurons grown in a minority with KO, may reveal differential abilities to develop or maintain synapses. We generated mixed co-cultures of 90% WT with 10% KO, and 90% KO with 10% WT, in comparison with pure WT and pure KO cultures. In one experiment, WT neurons were labeled with YFP and KO neurons were labeled with CFP (Fig. 2A). In a complementary experiment, labels were reversed (WT CFP and KO YFP) to ensure no bias due to YFP or CFP expression or differential nucleofection. GluN1 immunofluorescence was used to verify the genotype of YFP and CFP expressing neurons (Fig. 2B).

**Figure 3:**
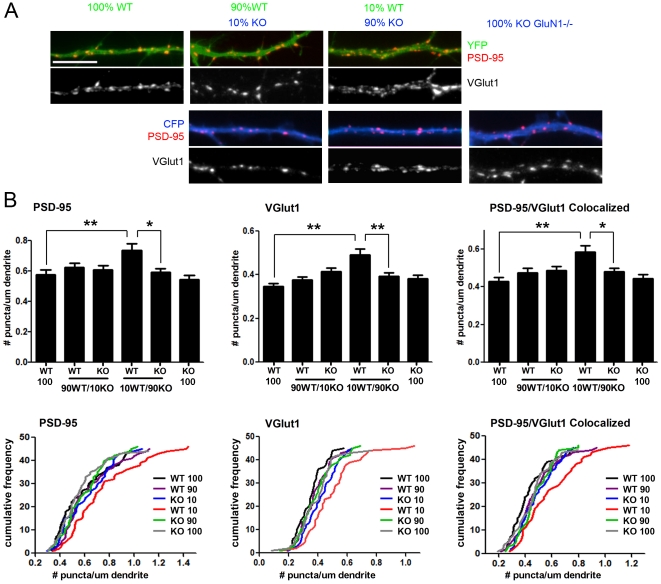
Synaptic competition: wild type neurons develop increased synapse density only when in a minority with predominantly GluN1 -/- neighbours. (A) Hippocampal neurons from littermate wild type or GluN1 -/- mice were labeled by nucleofection and grown in coculture as in Figure 2. At 14 DIV, neurons were fixed and immunolabeled for glutamatergic postsynaptic marker PSD-95 and presynaptic marker VGlut1. Neurons of defined genotype were identified by the YFP or CFP dendrite fill. (B) There was a significant difference among cocultures in density of PSD-95 puncta (ANOVA p = 0.0004), VGlut1 puncta (ANOVA p<0.0001), and colocalized PSD-95 and VGlut1 puncta marking excitatory synapses (ANOVA p<0.0001); n≥45 from 3 independent experiments. Posthoc Bonferroni's test comparing the 10% WT condition to neighbour 90% KO neurons and sister 100% WT neurons showed significant differences for PSD-95, VGlut1, and PSD-95/VGlut1 colocalized puncta (*p<0.01; **p<0.001). Complete pairwise posthoc Bonferroni's multiple comparison test revealed additional significant differences of the 10% WT condition to other conditions but no other significant differences. Data are presented as mean ± SEM (top) and cumulative frequency histograms (bottom).

Mixed genotype cultures of unequal composition were immunolabeled at 14 DIV for the glutamatergic postsynaptic scaffold PSD-95 family and the excitatory presynaptic vesicular glutamate transporter VGlut1 (Fig. 3A). Quantitation performed blind to genotype and culture composition revealed a significant difference among genotypes in these co-cultures in density of PSD-95 puncta, VGlut1 puncta, and PSD-95 puncta colocalized with VGlut1 marking excitatory synapses (Fig. 3B; ANOVA p = 0.0004 for PSD-95, p<0.0001 for VGlut1, and p<0.0001 for PSD-95 colocalized with VGlut1; n≥45 cells for each condition from 3 independent experiments). WT neurons in the 90% KO and 10% WT co-culture showed a 25% increase in PSD-95 puncta density, a 26% increase in VGlut1 puncta density, and a 22% increase in PSD-95/VGlut1 colocalized puncta density compared with KO neurons on the same coverslips (p<0.01 for PSD-95, p<0.001 for VGlut1, and p<0.01 for colocalized PSD-95/VGlut1 by Bonferroni's posthoc test). WT neurons in the 90% KO and 10% WT co-culture showed a 28% increase in PSD-95 puncta density, a 41% increase in VGlut1 puncta density, and a 36% increase in PSD-95/VGlut1 colocalized puncta density compared with sister pure WT cultures (p<0.001 for PSD-95, p<0.001 for VGlut1, and p<0.001 for colocalized PSD-95/VGlut1 by Bonferroni's posthoc test). Furthermore, in each of the 3 independent experiments comprising the dataset, WT neurons in the 90% KO and 10% WT coculture condition always showed the highest value among the 6 conditions for density of PSD-95, VGlut1, and colocalized PSD-95/VGlut1 puncta (ANOVA p = 0.016, p = 0.055, and p = 0.013 for individual experiments for colocalized PSD-95/VGlut1). In the combined data analyzed by full pairwise Bonferroni's multiple comparison test, the WT neurons in the 90% KO and 10% WT mix differed significantly from multiple other conditions, and no condition other than the WT neurons in the 90% KO and 10% WT mix showed any significant difference from any other condition. More specifically, the density of PSD-95, VGlut1, and PSD-95/VGlut1 colocalized puncta on KO neurons in the 90% WT and 10% KO co-culture did not differ significantly from WT neurons on the same coverslips or from sister pure KO cultures. Thus, in all 90∶10 mixed genotype co-culture combinations, only the minority WT neurons exhibited a consistent difference in steady state excitatory synapse density, out-competing their majority GluN1 -/- neighbours.

### Synaptic competition in GluN1 -/- and wild type co-culture requires NMDA receptor activity

**Figure 4:**
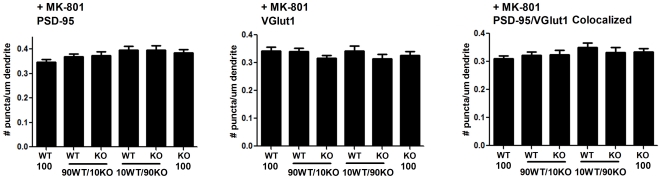
Synaptic competition in wild type and GluN1 -/- neuron co-culture is dependent on NMDA receptor activity. Hippocampal neurons from littermate wild type or GluN1 -/- mice were labeled by nucleofection and grown in coculture as in Figure 2, except that the NMDA receptor antagonist MK-801 was added chronically from DIV 0. At 14 DIV, neurons were fixed and analysed as in Figure 3. There was no significant difference among coculture conditions in density of PSD-95 puncta, VGlut1 puncta, and colocalized PSD-95 and Vglut1 puncta marking excitatory synapses; ANOVA p>0.1; n = 30 from 2 independent experiments (except n = 15 for 100% WT). Data are presented as mean ± SEM.

The enhanced innervation onto WT neurons in the 90% KO and 10% WT co-culture could result from two general mechanisms. One possibility is that activity and ion flux through functional NMDA receptor channels confers an advantage to the WT neurons compared with KO neighbours. The other possibility is that the physical presence of GluN1 and associated GluN2 [24] at synapses is sufficient to confer an advantage to WT neurons, for example, by stabilizing postsynaptic adhesion molecules through molecular interactions [25]. To differentiate between these possibilities, we repeated the 90% KO and 10% WT co-culture in the chronic presence of the NMDA receptor channel blocker MK-801. Chronic blockade of NMDA receptor channel activity abolished the effect of genotype on synapse density in 90∶10 mixed genotype co-cultures (compare Fig. 4 with Fig. 3B). In 90∶10 mixed genotype co-cultures treated chronically with MK-801, there was no significant difference among genotypes or culture composition in density of PSD-95 puncta, VGlut1 puncta, or PSD-95/VGlut1 colocalized puncta (Fig. 4; ANOVA p>0.1). Thus NMDA receptor channel activity is required for enhanced synapse density onto minority WT neurons compared to their majority GluN1 -/- neighbours.

## Discussion

We present here a cell culture paradigm of NMDA receptor-dependent synaptic competition. Wild type hippocampal neurons when cultured together with predominantly GluN1 -/- neighbours developed increased synapse density compared with their GluN1 -/- neighbours and compared with sister wild type neurons cultured alone. The physical presence of GluN1 was not sufficient to mediate this form of synaptic competition, NMDA receptor activity was required. The development of genotype-specific differences in synapse density was dependent on a defined ratio of genotypes in the circuit, occurring in a 10∶90 WT:KO co-culture, but not in a 50∶50 WT:KO co-culture or in a 90∶10 WT:KO co-culture. This simple model system may prove useful for defining rules and understanding mechanisms of NMDA receptor mediated synaptic competition.

**Figure 5:**
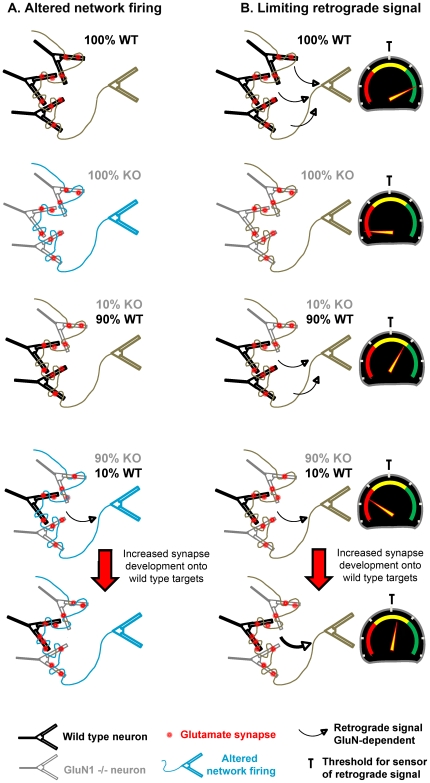
Potential mechanisms for the observed NMDA receptor-dependent synaptic competition. (**A**) Cultures with a majority of NMDA receptor-deficient neurons (100% KO, and 90% KO with 10% WT) may exhibit altered network firing patterns compared with cultures with a majority of NMDA receptor-competent neurons (100% WT, or 10% KO with 90% WT). The altered network firing may be specifically transduced in a manner dependent on NMDA receptor function into a retrograde signal that enhances synapse development. Thus, among all the culture conditions, only WT neurons in the 90% KO with 10% WT co-culture are subjected to the altered network firing and capable of transducing it to a retrograde signal to alter synapse development. (**B**) Synapses bearing functional NMDA receptors are proposed to generate a retrograde signal. In pure WT culture and co-cultures with a majority of WT neurons (10% KO with 90% WT), if each axon forms numerous synapses with WT neurons, total retrograde signal will be above a proposed threshold. In co-cultures with only a minority of WT neurons (90% KO with 10% WT), the total retrograde signal generated from initial random axon contact with WT neurons may not reach threshold. Additional synapses are selectively formed and/or stabilized onto WT neurons until the total retrograde signal to the presynaptic neuron reaches threshold. Since GluN1 -/- KO neurons in this mixed culture generate no retrograde signal, synapse development onto them is not changed. In pure GluN1 -/- KO cultures, no synapses are able to generate retrograde signal and thus there is no basis for altering synapse density. **Note**: Mechanisms (A) and (B) are not mutually exclusive, nor exhaustive; for example, more complicated scenarios may occur, such as selective synapse development according to both presynaptic and postsynaptic genotype.

Multiple mechanisms may contribute to the NMDA receptor-dependent synaptic competition observed here. One contributing factor may be altered network activity in cultures with predominantly NMDA receptor-deficient neurons compared with predominantly wild type neurons (Fig. 5A). These hippocampal neuron cultures grown in serum-free media with a separated glial feeder layer show irregular patterns of spontaneous activity [26], unlike the highly synchronized spontaneous oscillatory activity of neurons grown in serum on glia [26], [27], but perhaps more like hippocampal CA1 neurons *in vivo* (e.g. [28], [29]). However, in multiple culture systems and *in vivo*, genetic or pharmacological ablation of NMDA receptor activity alters network activity. Loss of NMDA receptor function typically reduces the amplitude or transforms the synchronous oscillatory activity into more irregular activity in culture [27], [30], perhaps also increasing AMPA receptor-mediated transmission [19], and reduces coordinated place-related firing in CA1 [31] and gamma frequency activity in striatum [32]. Thus, it is likely that the 90% KO and 100% KO cultures here may exhibit different patterns of network activity than the 90% WT and 100% WT cultures. It is possible that the WT neurons respond in an NMDA receptor-dependent way to the altered network activity in the 90% KO culture, through calcium signalling and perhaps differential gene expression, generating a cell surface or secreted retrograde signal that results in enhanced synapse density.

Other potential mechanisms independent of changes in network activity but also involving a retrograde signal could contribute to the observed NMDA receptor-dependent synaptic competition (Fig. 5B). A retrograde signal generated only by NMDA receptor-competent synapses could be sensed by each input neuron, and total retrograde signal compared with a target range. Only if the total retrograde signal does not reach a threshold following initial synapse development, then further selective synapse development may occur. In the simplest scenario, axons from each input neuron may randomly develop synapses onto neighbour neurons to a typical density (although there is also a possibility that presynaptic genotype may influence partner selection). If all, 90%, or even 50% of the synapses made by the input neuron were onto wild type neurons, the total retrograde signal would be sufficient to reach threshold. However, in the 10∶90 WT:KO co-culture, only 10% of synapses would randomly occur onto wild type neurons, insufficient for the total retrograde signal to the input neuron to reach threshold. Such neurons lacking sufficient total retrograde signal may then develop additional synapses onto NMDA receptor competent neurons to increase the total retrograde signal to reach threshold. In the pure GluN1 -/- culture, or in the 10∶90 WT:KO co-culture grown chronically in NMDA receptor channel blocker, threshold is not reached, but since no synapses are capable of generating retrograde signal there is no drive for increasing synapse density.

There are many candidates for potential secreted or cell surface retrograde signals that could function in such mechanisms. Local NMDA receptor activation might increase production or local secretion of classic messengers such as nitric oxide, endocannabinoids, or retinoic acid [33], [34], or of growth factor type signals such as BDNF and FGFs [35], [36], [37] or glial secretion of TNFα [38]. Alternately, local NMDA receptor activation might increase postsynaptic insertion or reduce endocytosis or alter conformation of surface proteins with trans-synaptic signalling capability. Neuroligins, LRRTMs, TrkC, NGLs, EphBs, ephrins, SynCAMs, NCAM, and cadherins are all candidates to convey such a signal [39], [40], [41], [42]. If a threshold-type sensor is involved (Fig. 5B), two key features, global summation and comparison with a threshold or target range, share commonalities with sensors for forms of homeostatic synaptic plasticity [43], [44]. BDNF and TNFα have been implicated as signals mediating both homeostatic synaptic plasticity [38], [45] and experience-dependent synaptic competition *in vivo* [46], [47]. The cadherin-β-catenin complex is also implicated in homeostatic synaptic plasticity [48], and NMDA receptor activity increases N-cadherin and associated β-catenin levels at the synapse [49], [50]. The more recently discovered cell surface synaptic organizing complexes have not been intensively studied in the context of activity-dependent synaptic modification. However, the potent ability of neuroligin or LRRTM binding to neurexins [39], or of TrkC or NGL-3 binding to PTPRs [41], [51], to trigger complex presynaptic differentiation suggests they may also generate far reaching signals for integration. Previous hippocampal culture experiments using mixed genotypes or transfection revealed activity-dependent effects on synapse density according to postsynaptic hyperpolarization (by expression of Kir2.1) [52], postsynaptic levels of BDNF [53] or CaMKII [54], [55], and presynaptic levels of synaptophysin [56]. At least some of these components may operate in the pathway triggered by differential presence of functional NMDA receptors.

With respect to cell biological mechanisms of the observed synaptic competition, input neurons may selectively increase their rate of synapse formation and/or reduce their rate of synapse elimination, only onto WT targets in the 10∶90 WT:KO co-culture. In wild type rat hippocampal cultures, NMDA receptor antagonists reduce rates of both synapse formation and synapse elimination [57]. In our competitive paradigm, perhaps reduced synapse elimination, i.e. enhanced stabilization rather than enhanced *de novo* formation, seems most likely, as in the classic model for studying synaptic competition, the neuromuscular junction [58]. However, differential synapse densities of silenced (by tetanus toxin light chain) versus active glutamatergic inputs onto retinal ganglion cells occurred by selective synapse formation rather than selective stabilization [59].

Perhaps it is surprising that GluN1 -/- neurons in 50∶50 or 10∶90 co-culture with wild type neurons exhibited normal synapse density with no reduction. These results are consistent with the finding of normal spine density following single cell in utero deletion of GluN1 in hippocampus *in vivo* [60]. However, other groups report reduced spine density with single cell deletion of GluN1 in cortical culture [19] or of GluN2B *in vivo* [17], and activity-independent reduction in spine and synapse density with GluN1 shRNA in hippocampal slice culture [61]. The effects of NMDA receptor subunit deletion on AMPA receptor mediated synaptic transmission are also complex [19], [60], [62], perhaps depending on synapse type, developmental stage, and environment.

More generally, NMDA receptor dependent synaptic competition in sensory systems is thought to involve selective stabilization of preferred inputs (inputs with greater activity or specific patterns of activity) and destabilization of non-preferred inputs. Our results suggest that the Hebbian ‘reward’ signal mediating selective formation or stabilization of preferred inputs may be distinct from the ‘punishment’ signal mediating selective reduction of non-preferred inputs. In our cell culture model of synaptic competition, wild type neurons were rewarded but GluN1 -/- neurons were not punished. Reduction of non-preferred synapses may require more demanding *in vivo* conditions such as limiting glial derived factors or higher neuron density altering the economics of synaptic competition.

Cell culture models testing rules and mechanisms of synaptic competition in defined circuits may contribute to a better understanding of the role of activity in sculpting nervous system connectivity during development. Targeted genetic modifications could be further combined with microfluidic chambers in which subsets of neurons can be pharmacologically manipulated [63] to aid in defining signalling pathways and temporal parameters mediating activity-regulated neuronal connectivity.

## Materials and Methods

### Ethics Statement

This study was conducted with approval of the University of British Columbia Animal Care Committee according to Protocol A09-0278.

### Cell Culture

GluN1 +/- mice were kindly provided by Drs. Michisuke Yuzaki and Tom Curran [10]. Genotyping was performed according to a previously described protocol [10]. Heterozygous mice were mated to obtain GluN1 -/- and littermate GluN1 +/+ wild type embryos. Hippocampi were dissected from individual 17–18 day embryos and stored overnight at 4°C in Hibernate E (Brain Bits) supplemented with B-27 (Invitrogen or Stemcell Technologies) pending genotyping of brainstem and tail tissue. Hippocampi were dissociated with papain (20 units/ml, 15 min, 37°C). Prior to plating, 5×10^5^ cells per dish were electronucleoporated using an Amaxa Nucleofector II (program O-005) with plasmids to express ECFP or EYFP from the CAG promoter consisting of the CMV immediate early enhancer and the chicken β-actin promoter [64], [65] (kind gift of S. Kaech and G. Banker with permission of J. Miyazaki for the CAG promoter). Nucleofected cells were plated onto poly-L-lysine treated glass coverslips in 60 mm petri dishes [64], for an effective estimated plating density of 3×10^5^ cells per dish. Neurons were maintained in Neurobasal medium (Invitrogen) supplemented with B-27 and 25 ug/ml bovine pancreatic insulin (Sigma-Aldrich) on glass coverslips inverted over a glial feeder layer. In the activity blockade experiments, 7.5 µM MK-801 (Enzo Life Sciences) NMDA receptor channel blocker was added chronically to culture media starting from DIV 0. MK-801 was reapplied during culture feeding on DIV 6 and DIV 12.

### Immunocytochemistry

For most experiments, neurons cultured on glass coverslips were fixed for 15 min in pre-warmed PBS with 4% paraformaldehyde and 4% sucrose followed by permeabilization with 0.25% Triton X-100 in PBS. For NR1 staining, coverslips were fixed and permeabilized in 4% paraformaldehyde/sucrose for 2 min followed by −20°C MeOH for 10 min followed by 0.04% Triton X-100 in PBS for 1 min. Fixed neuron cultures were blocked with 10% bovine serum albumin (BSA) in PBS (30 min, 37°C) prior to incubation with primary antibodies in PBS with 3% BSA (overnight, 37°C) and secondary antibodies (45 min, 37°C). Coverslips were washed six times for 2 min with PBS following each antibody incubation.

The following antibodies were used: anti-synapsin I (rabbit; polyclonal; 1∶1000; Millipore, AB1543P), anti-PSD-95 family (IgG2a; clone 6G6-1C9; 1∶1000; Thermo Fisher Scientific, MA1-045; recognizes PSD-95, PSD-93, SAP102, and SAP97), anti-VGlut1 (guinea pig; polyclonal; 1∶4000; Millipore; AB5905), anti-MAP2 (chicken, polyclonal; 1∶10,000; AbCam, ab5392) and anti-NR1 (mouse IgG; CT; 1∶1000; Millipore, 05–432 and IgG2a; clone 54.1; 1∶1000; Invitrogen, 32–0500). Secondary antibodies were highly cross-adsorbed antibodies mainly generated in goat: Alexa-568 anti-rabbit, Alexa-568 anti-IgG2a, Alexa-647 anti-guinea pig, Alexa-568 anti-pan-mouse (1∶500; Invitrogen), and AMCA conjugated anti-chicken IgY (donkey IgG; 1∶200; Invitrogen).

### Imaging and quantitative fluorescence analysis

All imaging and analysis was done blind to cell genotype and culture composition. Images were acquired on a Zeiss Axioplan2 microscope with a 63X 1.4 numerical aperture oil objective and Photometrics Sensys cooled CCD camera using MetaVue imaging software (Molecular Devices) and customized filter sets. Individual antibodies were tested with single colour secondary staining to confirm no detectable bleed through between channels AMCA, CFP, YFP, Alexa-568, and Alexa-647. Images in each channel were acquired in grey scale from individual channels using the same exposure time across all cells, and pseudo-colour overlays for presentation were prepared using Adobe Photoshop. Healthy CFP- and YFP-positive cells were randomly selected for imaging and 2–3 dendrites chosen by fluorescent protein fill for quantitative analysis. Synaptic marker channels were thresholded by intensity and puncta per dendrite length counted. To define colocalized objects, thresholded puncta from the VGlut channel were dilated by 2 pixels and compared for pixel overlap with thresholded puncta from the PSD-95 channel. Statistical analyses were performed with GraphPad Prism.
